# Longitudinal relationship between traumatic brain injury and the risk of incident optic neuropathy: A 10-year follow-up nationally representative Taiwan survey

**DOI:** 10.18632/oncotarget.21008

**Published:** 2017-09-18

**Authors:** Ying-Jen Chen, Chang-Min Liang, Ming-Cheng Tai, Yun-Hsiang Chang, Tzu-Yu Lin, Chi-Hsiang Chung, Fu-Huang Lin, Chang-Huei Tsao, Wu-Chien Chien

**Affiliations:** ^1^ Department of Ophthalmology, Tri-Service General Hospital, National Defense Medical Center, Taipei, Taiwan, R.O.C; ^2^ Graduate Institute of Medical Sciences, National Defense Medical Center, Taipei, Taiwan, R.O.C; ^3^Department of Ophthalmology, Shuang Ho Hospital, Taipei Medical University, New Taipei City, Taiwan, R.O.C; ^4^ School of Public Health, National Defense Medical Center, Taipei, Taiwan, R.O.C; ^5^ Department of Medical Research, Tri-Service General Hospital, National Defense Medical Center, Taipei, Taiwan, R.O.C; ^6^ Department of Microbiology and Immunology, National Defense Medical Center, Taipei, Taiwan, R.O.C

**Keywords:** traumatic brain injury, optic neuropathy, national health insurance research database

## Abstract

Accumulating evidences had shown that traumatic brain injury was associated with visual impairment or vision loss. However, there were a limited number of empirical studies regarding the longitudinal relationship between traumatic brain injury and incident optic neuropathy. We studied a cohort from the Taiwanese National Health Insurance data comprising 553918 participants with traumatic brain injury and optic neuropathy-free in the case group and 1107836 individuals without traumatic brain injury in the control group from 1st January 2000. After the index date until the end of 2010, Cox proportional hazards analysis was used to compare the risk of incident optic neuropathy. During the follow-up period, case group was more likely to develop incident optic neuropathy (0.24%) than the control group (0.11%). Multivariate Cox regression analysis demonstrated that the case group had a 3-fold increased risk of optic neuropathy (HR = 3.017, 95% CI = 2.767–3.289, *p* < 0.001). After stratification by demographic information, traumatic brain injury remained a significant factor for incident optic neuropathy. Our study provided evidence of the increased risk of incident optic neuropathy after traumatic brain injury during a 10-year follow-up period. Patients with traumatic brain injury required periodic and thorough eye examinations for incident optic neuropathy to prevent potentially irreversible vision loss.

## INTRODUCTION

Traumatic brain injury (TBI) had been proposed as a main risk factor of morbidity and mortality worldwide, with an estimated incidence ranging from 106 to 790 per 100000 people per year, and lead to subsequent neurological sequela and devastating disabilities [1]. A comprehensive systematic review and meta-analysis of the worldwide incidence of TBI also showed that the pooled annual incidence proportion for all ages was 295 per 100,000 (95% confidence interval: 274–317) [2]. The mild form of TBI had gained great importance due to extended awareness of the potential long-term detrimental effects of repeated concussive events, such as depressive disorder, cognitive impairment, neurodegenerative diseases, and a negative impact on multiple aspects of vision [3]. Patients with mild TBI might have abnormalities in saccades, pursuit, convergence, accommodation, and the vestibulo-ocular reflex, while moderate and severe TBI patients might have additional issues, such as ocular motor palsies, optic neuropathies, and orbital pathologies [4]. Depending on its location and severity, TBI could contribute to structural and functional damage to the cranial nerves, optic nerve tract or other circuitry, and occipital lobe [5].

Over the past 20 years, visual impairment elicited by TBI instead of eye disorders has been the focus of increasing attention. Optic neuropathy, a cause of vision loss in patients with TBI, had diverse underlying mechanisms and typically manifest with decreased visual acuity, altered color vision, and abnormal visual field in the affected eye [6]. Major traumatic brain injury had been associated with traumatic optic neuropathy, which affected from 40 to 72% of individuals with TBI [7]. Traumatic optic neuropathy (TON) commonly occurred following TBI and was characterized by vision loss along with no obvious evidence of damage to the eye and possibly the optic nerve [8, 9]. Many aspects of visual functions related to higher cognitive function were characteristically compromised in TBI, which may often give rise to a delay in diagnosis and irrevocably cause visual loss [4]. Carta and his colleagues had offered four negative prognostic factors to evaluate the visual prognosis in cases of optic neuropathy following head trauma, including 40 years old or more, unconsciousness, poor response after steroid therapy, and the existence of blood around the posterior ethmoid cells [10]. However, there was substantial controversy concerning the prognostic impact and pathophysiology of traumatic optic neuropathy. While there was a strong association between TBI and optic neuropathy, little empirical evidence had been found to determine the longitudinal effect of TBI on optic neuropathy. Therefore, the aim of our study was to determine the trajectory of incident optic neuropathy after TBI and identify the profile of prognostic factors.

## RESULTS

**Figure 1:**
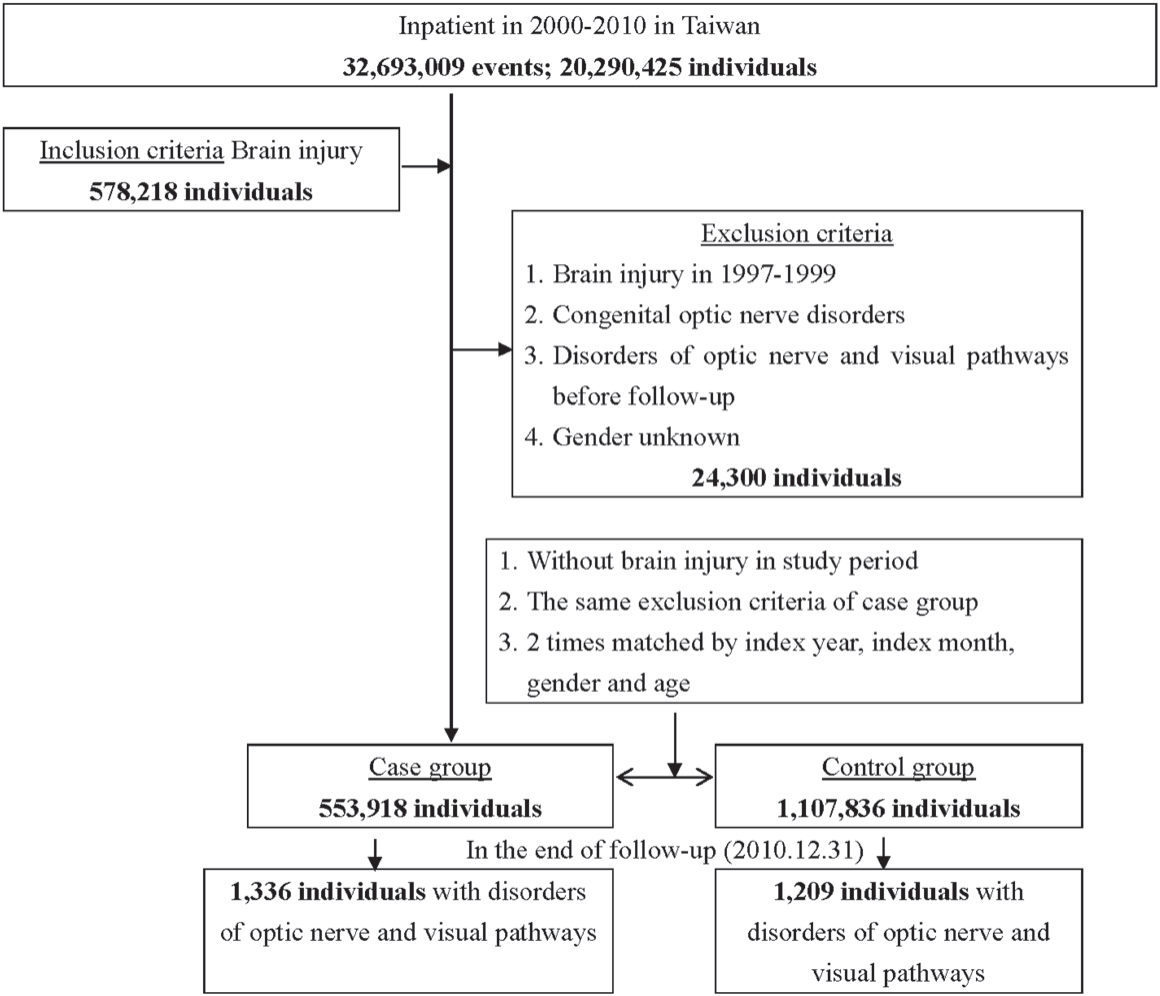
The flowchart of study sample selection

Of the total sample, 578,218 patients were diagnosed with brain injury. After excluding unknown gender, past histories of brain injury, congenital optic nerve disorders, and optic neuropathy from 1997–1999, 553,918 patients with brain injury were identified as the case group. We used matching age and sex to randomly select 1,107,836 individuals as the control group (1:2). The flowchart of study sample selection from the Longitudinal Health Insurance Database (LHID) was shown in Figure 1.

The detailed distributions of the demographic data in the case group and control group was provided in Table 1. After matching for age and gender, the case group was more likely to have low income, intentional injury, residence in the less urbanized areas, and low health care level at the time of the index date. The mean ages of the case group and control group were 42.06 and 41.60 years, respectively.

**Table 1:** Characteristics of study in the baseline

Variables	Total		Brain injury				*P* value
		With (Case)		Without (Control)			
	*n*	%	*n*	%	*n*	%		
**Total**	1,661,754		553,918	33.33	1,107,836	66.67	
**Gender**							0.999
Male	1,024,038	61.62	341,346	61.62	682,692	61.62	
Female	637,716	38.38	212,572	38.38	425,144	38.38	
**Age (years)** **(mean ± SD)**	41.92±24.50		42.06±24.45		41.60±25.45		0.873
**Low-income**							<0.001
Without	1,641,250	98.77	545,533	98.49	1,095,717	98.91	
With	20,504	1.23	8,385	1.51	12,119	1.09	
**CCI (mean ± SD)**	0.51±1.48		0.16±0.61		0.68±1.74		< 0.001
**Intentionality of injury**							< 0.001
Unintentional injury	427,738	93.91	352,283	93.56	75,455	95.56	
Intentional injury	27,749	6.09	24,241	6.44	3,508	4.44	
**Season**							< 0.001
Spring (April-May)	431,510	25.97	137,314	24.79	294,196	26.56	
Summer (June-August)	406,582	24.47	136,934	24.72	269,648	24.34	
Autumn (September-November)	386,686	23.27	140,382	25.34	246,304	22.23	
Winter (December-Feburary)	436,976	26.30	139,288	25.15	297,688	26.87	
**Urbanization level**							< 0.001
High	515,319	31.01	128,002	23.11	387,317	34.96	
Middle	701,371	42.21	225,988	40.80	475,383	42.91	
Low	445,064	26.78	199,928	36.09	245,136	22.13	
**Level of care**							< 0.001
Hospital center	490,277	29.50	114,666	20.70	375,611	33.90	
Regional hospital	621,198	37.38	231,041	41.71	390,157	35.22	
Local hospital	550,279	33.11	208,211	37.59	342,068	30.88	

Unintentional injury: ICD-9-CM E800-E949; Intentional injury: ICD-9-CM E950-E979, E990-E999.

SD, standard deviation; CCI, Charlson Comorbidity Index.

The distributions of incident optic neuropathy and related clinical manifestations for two groups during the 10-year follow-up period were presented in Table 2. Compared with the control group (0.11%), the case group was more likely to develop incident optic neuropathy (0.24%) during the follow-up period (*P* < 0.001).

**Table 2:** Characteristics of study in the end of follow-up

Variables			Brain injury				*P* value
	Total		With (Case)		Without (Control)		
	*n*	%	*n*	%	*n*	%	
**Incident optic neuropathy**							< 0.001
Without	1,659,209	99.85	552,582	99.76	1,106,627	99.89	
With	2,545	0.15	1,336	0.24	1,209	0.11	
**Catastrophic illness**							< 0.001
Without	1,445,313	86.98	501,531	90.54	943,782	85.19	
With	216,441	13.02	52,387	9.46	164,054	14.81	
**CCI (mean±SD)**	0.88 ± 2.29		0.47 ± 1.50		1.08 ± 2.58		< 0.001
**Intentionality of injury**							< 0.001
Unintentional injury	321,114	94.49	246,209	94.16	74,905	95.60	
Intentional injury	18,723	5.51	15,274	5.84	3,449	4.40	
**Surgery**							< 0.001
Without	1,038,767	62.51	381,378	68.85	657,389	59.34	
With	622,987	37.49	172,540	31.15	450,447	40.66	
**Length of days** **(mean ± SD)**	17.42 ± 19.78		17.03 ± 18.78		17.61 ± 20.23		< 0.001
**Medical costs (NT$) (mean ± SD)**	147,927.90 ± 188,290.34		144,274.50 ± 177,967.85		149,754.96 ± 192,968.37		< 0.001
**Prognosis**							< 0.001
Survive	1,533,440	92.28	518,426	93.59	1,015,014	91.62	
Mortality	128,314	7.72	35,492	6.41	92,822	8.38	

Unintentional injury: ICD-9-CM E800-E949; Intentional injury: ICD-9-CM E950-E979, E990-E999.

SD, standard deviation; CCI, Charlson Comorbidity Index.

**Figure 2:**
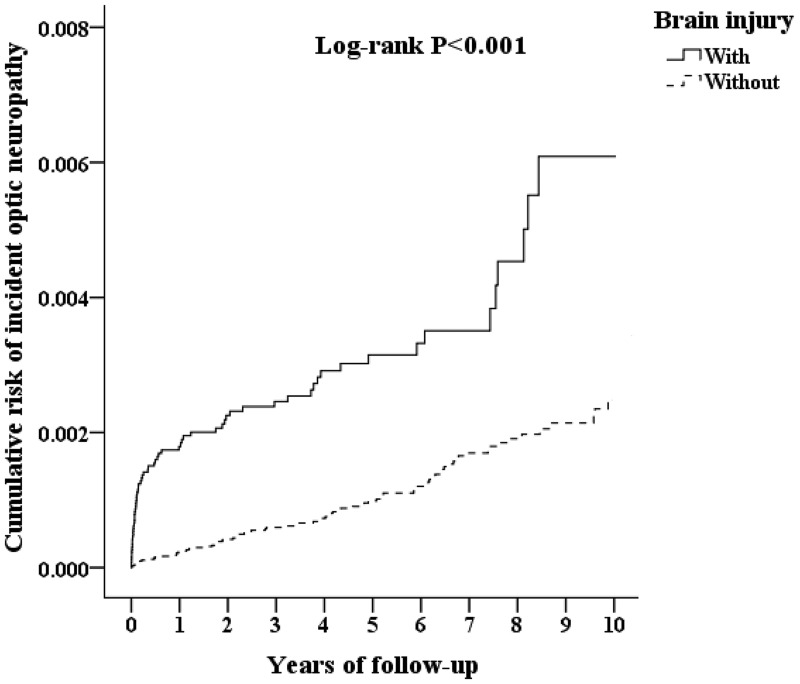
Kaplan Meier plot of the cumulative risk of incident optic neuropathy in patients with brain injury

A Kaplan-Meier graph of the cumulative risks of incident optic neuropathy was shown in Figure 2, and the log-rank test revealed that the case group had significantly higher cumulative risks than the control group (*p* < 0.001). The crude hazard ratio and adjusted hazard ratio of incident optic neuropathy in the two groups were presented in Table 3. For the crude hazard ratio of the case group, our findings addressed that the risk of incident optic neuropathy was 3.360 times of the control group during the follow-up period. Notably, the risk of incident optic neuropathy among the male patients with brain injury was significantly higher than female patients with brain injury, by a multiple of 1.463. The adjusted hazard ratios of incident optic neuropathy within the 10-year follow-up were 3.017 (95% CI = 2.767–3.289) times greater for patients with brain injury than those for patients without brain injury. Furthermore, male patients with brain injury had a greater likelihood of the developing optic neuropathy (*p* < 0.001, 95% CI= 1.253–1.487). Brain injury was associated with an increased risk of incident optic neuropathy (Table 4). The patients with brain injury had a 3.017-fold increased risk of incident optic neuropathy, while patients with brain injury and low income had a 3.468-fold increased risk of incident optic neuropathy.

**Table 3:** Factors of incident optic neuropathy in the end of follow-up by using Cox regression

Variables	Crude HR	95% CI	95% CI	*P* value	Adjusted HR	95% CI	95% CI	*P* value
**Brain injury**								
Without (Control)	Reference				Reference			
With (Case)	3.360	3.102	3.638	<0.001	3.017	2.767	3.289	< 0.001
**Gender**								
Male	1.463	1.344	1.593	<0.001	1.365	1.253	1.487	< 0.001
Female	Reference				Reference			
**Age (years)**	0.993	0.992	0.995	<0.001	0.996	0.994	0.998	< 0.001
**Low-income**								
Without	Reference				Reference			
With	1.302	1.028	1.649	0.028	1.461	1.152	1.852	0.002
**Catastrophic illness**								
Without	Reference				Reference			
With	0.949	0.852	1.057	0.340	1.234	1.096	1.389	0.001
**CCI**	0.834	0.808	0.861	<0.001	0.833	0.805	0.863	< 0.001
**Intentionality of injury**								
Unintentional injury	Reference				Reference			
Intentional injury	1.125	1.005	1.612	0.039	1.010	1.002	1.549	0.048
**Season**								
Spring (April-May)	Reference				Reference			
Summer (June-August)	0.889	0.796	0.992	0.036	0.869	0.778	0.971	0.013
Autumn (September-November)	0.878	0.787	0.978	0.019	0.837	0.750	0.933	0.001
Winter (December-Feburary)	0.914	0.818	1.021	0.112	0.914	0.818	1.022	0.115
**Urbanization level**								
High	1.734	1.549	1.942	< 0.001	0.850	0.740	0.975	0.020
Middle	1.599	1.436	1.781	< 0.001	0.969	0.857	1.095	0.610
Low	Reference				Reference			
**Level of care**								
Hospital center	5.808	5.017	6.724	< 0.001	8.194	6.964	9.641	< 0.001
Regional hospital	2.754	2.367	3.204	< 0.001	2.884	2.475	3.361	< 0.001
Local hospital	Reference				Reference			
**Surgery**								
Without	Reference				Reference			
With	0.871	0.802	0.945	0.001	0.700	0.644	0.762	< 0.001
**Length of days**	1.008	1.007	1.009	<0.001	1.007	1.006	1.008	< 0.001
**Medical costs (NT$)**	1.000	1.000	1.001	<0.001				

Unintentional injury: ICD-9-CM E800-E949; Intentional injury: ICD-9-CM E950-E979, E990-E999.

Medical cost had collinearity with length of days. HR, hazard ratio; CI, confidence interval; Adjusted HR, adjusted variables listed in the table.

**Table 4:** Factors of incident optic neuropathy in the end of follow-up stratified by variables listed in the table by using Cox regression

Variables	Brain injury						Ratio	Adjusted HR	95%CI	95%CI	*P* value
	With (Case)			Without (Control)							
	Event	PYs	Rate (per 105 PYs)	Event	PYs	Rate (per 105 PYs)					
**Total**	1,336	910,651.73	146.71	1,209	3,650,280.65	33.12	4.429	3.017	2.767	3.289	< 0.001
**Gender**											
Male	942	540,396.06	174.32	848	2,275,466.21	37.27	4.677	3.001	2.707	3.326	< 0.001
Female	394	370,255.67	106.41	361	1,374,814.44	26.26	4.053	3.071	2.620	3.599	< 0.001
**Age (years)**	1,336	910,651.73	146.71	1,209	3,650,280.65	33.12	4.429	3.017	2.767	3.289	< 0.001
**Low-income**											
Without	1,301	885,069.18	146.99	1,173	3,562,625.97	32.93	4.464	3.003	2.751	3.278	< 0.001
With	35	25,582.55	136.81	36	87,654.67	41.07	3.331	3.468	2.108	5.705	< 0.001
**Catastrophic illness**											
Without	1,131	783,487.97	144.35	1,019	2,955,425.49	34.48	4.187	2.966	2.700	3.257	< 0.001
With	205	126,803.76	161.67	190	694,855.16	27.34	5.912	3.677	2.956	4.573	< 0.001
**CCI**	1,336	910,651.73	146.71	1,209	3,650,280.65	33.12	4.429	3.017	2.767	3.289	< 0.001
**Intentionality of injury**											
Unintentional injury	729	147,192.25	495.27	70	274,202.33	25.53	19.401	9.015	6.921	11.741	< 0.001
Intentional injury	43	7,826.35	549.43	8	9,875.35	81.01	6.782	3.869	1.696	8.825	0.001
**Season**											
Spring (April-May)	337	195,829.10	172.09	301	829,185.91	36.30	4.741	3.300	2.778	3.920	< 0.001
Summer (June-August)	323	232,489.20	138.93	302	929,267.31	32.50	4.275	3.119	2.624	3.708	< 0.001
Autumn (September-November)	386	273,395.14	141.19	290	1,028,489.08	28.20	5.007	3.273	2.764	3.874	< 0.001
Winter (December-Feburary)	290	208,938.30	138.80	316	863,338.36	36.60	3.792	2.523	2.112	3.013	< 0.001
**Urbanization level**											
High	437	220,718.66	197.99	453	1,100,766.45	41.15	4.811	2.964	2.565	3.424	< 0.001
Middle	594	379,414.13	156.56	606	1,635,079.35	37.06	4.224	3.027	2.671	3.431	< 0.001
Low	305	310,518.94	98.22	150	914,434.85	16.40	5.988	3.429	2.761	4.259	< 0.001
**Level of care**											
Hospital center	622	216,564.07	287.21	802	1,204,301.98	66.59	4.313	2.646	2.360	2.967	< 0.001
Regional hospital	588	422,275.10	139.25	328	1,593,946.02	20.58	6.767	3.855	3.317	4.481	< 0.001
Local hospital	126	271,812.56	46.36	79	852,032.65	9.27	5.000	3.091	2.259	4.228	< 0.001
**Surgery**											
Without	841	559,357.80	150.35	842	2,316,196.29	36.35	4.136	2.701	2.423	3.012	< 0.001
With	495	351,293.93	140.91	367	1,334,084.36	27.51	5.122	3.653	3.159	4.225	< 0.001

Unintentional injury: ICD-9-CM E800-E949; Intentional injury: ICD-9-CM E950-E979, E990-E999.

PYs, Person-years; Ratio, Rate in case ÷ Rate in control; Adjusted HR, Adjusted Hazard ratio, Adjusted for all the variables above; CI, confidence interval.

## DISCUSSION

The fact that TBI could provoke optic neuropathy was already well recognized, but there have been few attempts to explore the risk factors associated with optic neuropathy and establish the longitudinal relationship between TBI and incident optic neuropathy. Using a population-based dataset, our study demonstrated that a significant positive relationship existed between TBI and incident optic neuropathy even after adjusting for the patients’ demographic characteristics, comorbidities, and clinical pertinent covariates. During a 10-year follow-up period, compared with the control group, patients in the case group had an increased risk of incident optic neuropathy with an overall adjusted HR of 3.017 (95% CI 2.767–3.289). Notably, the cumulative risk of incident optic neuropathy in the case group was obviously higher than that in the control group during the 8 to 9 years after the initial diagnosis of TBI.

During the first six months after brain injury, our study demonstrated that the cumulative risk of incident optic neuropathy in the case group was higher than that in the control group. One plausible explanation for this observation was that incident optic neuropathy following the brain injury was attributed to indirect TON. Surveillance studies of TON for pediatric and adult populations in England showed that the overall incidence of TON was approximately 1/million people in both adults and children [11, 12]. The most commonly depicted form of TON is caused by indirect injury to the optic nerve. The incidence of indirect TON had been addressed in 0.5–8.0% of cases of head trauma [13, 14]. Indirect TON was typically attributed to the transmission of concussive forces to the optic canal a result of blunt head trauma or brain injury. Forehead injury but not temporal region injury may contribute to blindness along with a loss of consciousness [15]. A previous histopathological analysis revealed significant hemorrhage in the optic nerve sheath and the nerve interstitium associated with shearing lesions and ischemic necrosis of the intracanalicular and intracranial segments of the nerve [5, 16]. Impairment in retinal blood circulation was a critical factor that produced optic neuropathy and was associated with axonal injury [17, 18]. In one retrospective study concerning visual function deficits and TBI, the investigators showed that patients with moderate or severe TBI often exhibited complications with cranial nerve lesions, such as the intracanalicular portion of the optic nerve, the oculomotor nerve exit from the midbrain, the midbrain exit site of the trochlear nerve, and the extradural space of the abducens nerve [19]. Following optic nerve injury, significant descending degeneration of the retinal ganglion cells does not occur until approximately three weeks following injury, with maximal loss by six weeks [20]. Consistent with the findings obtained in our study, the cumulative risk of TON in the case group was overtly higher than that in the control group at the initial follow-up period. It was tempting to speculate that the anatomical disruption and mechanical compression from TBI contributed to the resulting injury of the optic nerve, including hematoma, edema, vascular insufficiency [21].

It is of interest that increasing cumulative risks of incident optic neuropathy in the case group were noted during the 8 to 9 years after initial diagnosis of TBI. There were a number of plausible explanations for this increased risk. The intracanalicular portion of the optic nerve was the most susceptible to posterior indirect optic damage which was followed by shearing and ischemia that may have a long-term effect on optic nerve damage and vision loss [22]. The acceleration and deceleration injuries produced by TBI may cause the damage to the optic nerve, including nerve fiber tears, shearing of dural vessels, vasospasm, vascular compromise, and ischemia [23]. During the partial ischemia and reperfusion of transiently ischemic regions, intermittent hypoxia promotes production of reactive oxygen species, which increased oxidative stress, activated systemic inflammation, and lead to reperfusion damage and decreased bioavailability of endothelial nitric oxide [24, 25]. Vascular dysregulation due to nitric oxide/endothelin imbalance or abnormal platelet aggregation also has indirect effects on optic nerve head blood flow [26]. In patients with TBI complicated with subdural or epidural hematomas, disruption of the blood–brain barrier allowed access of cytokines and inflammatory cells to central nervous system compartments preceding the loss of autoregulation caused by fluctuation of intracranial pressure [7]. Despite the normal appearance of the optic nerve, microscopic studies might reveal several pathological processes, such as chronic inflammation with phagocytosis, myelin degeneration, axon loss, and microcirculatory alterations. Trauma could precipitate a destructive cascade of interrelated events that caused delay secondary damage within the central nervous system [27]. These events include oxidative stress, release of inflammatory mediators, and intracellular calcium influx leading to excitotoxic damage and apoptosis [28, 29]. In addition, animal models of mild TBI in mice revealed single mild TBI caused traumatic axonal injury in the optic nerve/tract, cerebellum, corticospinal tract, lateral lemniscus and corpus callosum, and single injury were associated with retinal ganglion cell loss and optic nerve degeneration [30]. It was possible that TBI insulted the blood-brain barrier and the ensuing Inflammatory cell migration resulted in delayed recovery that harbored a predisposing milieu for TON. This issue warranted further prospective investigation to delineate the pathophysiological mechanisms and subsequent strategies to minimize the risk of optic neuropathy.

Our results needed to be interpreted within the context of the following limitations. First, the NHI dataset only had medical claim without detailed clinical information regarding the visual acuity, which failed to follow up the recovery status of visual acuity. Second, patients diagnosed with TBI and incident optic neuropathy were identified according to the insurance claims data without image data. The severity of the injuries was not available because of the lack of detailed clinical information in the ICD-9 coding system. Third, although we had done our best to adjust for the influence of socioeconomic status there were several confounding factors for TBI and incident optic neuropathy, such as smoking history, alcohol consumption, nutrition, and body mass index. Fourth, the current study explored the connection of indirect TON and TBI only for single TBI events (as patients with prior history of TBI were excluded). However, several animal model data and clinical studies indicated that repeated TBI events may have much more profound and lasting (chronic) effects on the visual system, including optic nerve inflammation and degeneration [30–32].

Our study provided evidence that patients with TBI were at a higher risk of incident optic neuropathy than those without TBI during a 10-year follow-up period regardless of controlling for demographic factors and comorbidities. Following the TBI, meticulous ophthalmological evaluations were recommended to clarify the nature of optic neuropathy at regular intervals for up to 8∼9 years. Therefore, early recognition by thorough examinations with raised awareness in the clinical setting could preserve visual function and prevent a catastrophic disability.

## MATERIALS AND METHODS

### Data and study population

Our study data were representative of the Taiwanese population from the Taiwan national health insurance (NHI) program. Taiwan adopted a national health insurance system on March 1, 1995, which was a government administered insurance-based national healthcare system. The NHI program enrolled 99.9% of Taiwan residents in 2014 [33]. The National Health Research Institutes (NHRI) and National Health Insurance Administration used a systematic sampling method to randomly assemble a database of 1000000 insurants from the year 2000 registry, which was called the LHID. This database of this program contains registration files and original claim data for reimbursement during the 10-year follow-up period (from January 1st, 2000 to December 31st, 2010). To ensure confidentiality, all personal identification was encrypted and deleted before the data were provided to the researchers based on the assurance of confidentiality. NHRI provided the basic demographics and health care costs between the sample group, and there were no significant differences among the participants [34]. We used comprehensive information from the hospitalization of LHID, including gender, age, date of admission, date of discharge, date of visit, comorbidity, level of care, survival, diagnosis of discharge and outpatient visit (by International Classification of Diseases, Ninth Revision, Clinical Modification (ICD-9-CM) coding system). Because data related to individual identification in the LHID were encrypted and remained anonymous during the whole process by NHRI, our study was approved to meet the criteria for exemption by the Institutional Review Board (IRB) of the Tri-Service General Hospital (TSGH) (the approval number TSGH-IRB No. 2-104-05-126). Moreover, the IRB waived the informed consent requirement.

### Definition of optic neuropathy

The primary outcome of our study was incident optic neuropathy. Diagnosis of optic neuropathy was identified from the admission and inpatient claim database based on ICD-9-CM codes 377 (Disorders of optic nerve and visual pathways), 377.1 (Optic atrophy), 377.2 (Other disorders of optic disc), 377.3 (Optic neuritis), 377.39 (Other optic neuritis), 377.4 (Other disorders of optic nerve), 377.41 (Ischemic optic neuropathy), 377.49 (Other compression of optic nerve), 377.9 (unspecified disorder of optic nerve and visual pathways), 950 (injury to optic nerve and pathways), 950.0 (optic nerve injury), or 950.9 (Injury to unspecified optic nerve and pathways). To select accurate enrollment of incident optic neuropathy, these patients were required to have pertinent ophthalmological examinations on the day of diagnosis, including visual field examination and fundus examinations. In addition, We excluded patients with hereditary optic atrophy (ICD-9-CM 377.16) to reduce any influence on trauma-related optic neuropathy.

### Study design and patients inclusion

This population-based cohort study composed of all patients with a first-time diagnosis of brain injury (ICD-9 800–804 and 850–854) during the period of January 1, 2000 to December 31, 2010. These patients were evaluated for clinical manifestations and followed from the first index date until the event. After analyzing the LHID with antecedent data from January 1, 1997, we excluded subjects with optic neuropathy or brain injury during the period to 31st December 1999.

### Study variables

Associated comorbidities were evaluated by the Charlson comorbidity index (CCI) according to the diagnoses recorded in the NHI, including hypertension (ICD-9-CM: 401–405), diabetes mellitus (ICD-9-CM: 250), chronic kidney disease (ICD-9-CM: 585), chronic obstructive pulmonary disease (ICD-9-CM: 490–496), ischemic heart disease (ICD-9-CM: 410–414), congestive heart disease (ICD-9-CM: 428–429), malignancy (ICD-9-CM: 140–208), and stroke (ICD-9-CM: 430–438). The socio-environmental factors and clinical information included income (the presence of low-income), hospital levels (medical center, regional and local hospital), seasons, urbanization level, surgery, length of days, medical costs, and intentionality of injury. The presence of low income was based on a monthly income < 18,000 New Taiwan dollars according to the Ministry of Health and Welfare of Taiwan [35]. In Taiwan’s NHI program, patients with any of the 30 categories of catastrophic illness specified by the Bureau of the NHI can apply for catastrophic illness certificates and any insurant with major diseases such as cancer can apply for a catastrophic illness certification [36]. The urbanization levels of the city in Taiwan were categorized into seven levels, with level 1 referring to the “most urbanized” and level 7 referring to the “least urbanized” communities. The assessment criteria of urbanization level included the population density of the residential area, population ratio of elderly people, number of agricultural workers, educational level, and number of physicians per 100,000 people. Urbanization in our study was stratified into three levels, including high, middle, and low [37].

### Data analysis

All statistical analysis of data was performed using the Statistical Product and Service Solutions 22nd edition (Armonk, NY: IBM Corp.). Chi-square test or Fisher’s exact test was carried out to examine the differences in categorical data, and *t*-test was conducted with continuous data. Two-sided *p*-values of less than 0.05 were regarded as statistically significant for this study. Cumulative risks of optic neuropathy were calculated with the Kaplan-Meier method and compared by log-rank test. To account for the association between the brain injury *and incident optic neuropathy,* we used multivariate Cox proportional hazard models while adjusting for pertinent clinical characteristics.
